# A stakeholders’ pathway towards a future land use and food system in Germany

**DOI:** 10.1007/s11625-022-01212-0

**Published:** 2022-09-01

**Authors:** Livia Rasche, Uwe A. Schneider, Jan Steinhauser

**Affiliations:** 1 Research Unit Sustainability and Climate Risks, Universität Hamburg, Grindelberg 5, 20144 Hamburg, Germany; 2 Energy, Climate, and Environment Program, International Institute for Applied Systems Analysis (IIASA), Schlossplatz 1, 2361 Laxenburg, Austria

**Keywords:** FABLE calculator, Food system, GHG emissions, Land use change, Stakeholder survey, Sustainable transformation

## Abstract

Food systems contribute considerably to greenhouse gas (GHG) emissions and influence land use. In Germany, many strategies have been proposed by policy-makers to reduce negative impacts and make the food system more sustainable. It is unclear how close the suggested policies, when bundled, will bring the food and land use system towards the targeted goals; and what stakeholders from non-policy-making organizations consider realistic changes in the German food system. We thus surveyed different stakeholder groups on their opinions about realistic changes in the food and land use system in Germany up to 2050, developed four stakeholder pathways, and used an accounting tool to determine the effect of each pathway on indicators such as land use, GHG emissions, and biodiversity conservation potential. The assessment showed that GHG emissions from agricultural activities and land use are reduced from 66 to − 2–22 TgCO_2_e by 2050, while the area where natural processes predominate increases from 19 to 27–32%, and the resilience of the food system is not negatively influenced. The change is caused mainly by a diet-change-induced reduction of livestock production and agricultural area transformation into areas with higher carbon sequestration rates. If followed, the common stakeholder pathway (based on all stakeholder responses) would thus lead towards a sustainable food and land use system, but only if the underlying assumption of a drastic diet change towards more plant-based products comes true. Stakeholders from the academic and public sectors were more likely to assume that such a change was realistic than stakeholders from the private sector.

## Introduction

The European Union aims to make Europe the first climate-neutral continent of the world.^1^ The pathway towards this goal is outlined in the European Green Deal (European Comission 2019), which includes supplying clean, affordable and secure energy, mobilizing the industry for a clean and circular economy, building and renovating in an energy and resource-efficient way, zero pollution, preserving and restoring ecosystems and biodiversity, accelerating the shift to sustainable smart mobility, and building a fair, healthy and environmentally friendly food system. Concrete targets in the pursuit of these goals comprise a reduction of greenhouse gas (GHG) emissions by at least 55% by 2030, a renewable energy share of at least 40%, an increase in energy efficiency of 36–39% compared to 1990, a removal of 310 Tg carbon by sequestration in restored forests, soils, wetlands and peatlands, and the extension of the EU-wide network of protected areas to cover at least 30% of land and 30% of sea. In Germany, the parliament went beyond EU targets, aiming at an overall 65% reduction of GHG emissions by 2030 compared to 1990 levels, and 88% by 2040, reaching net-zero emissions by 2045 and net-negative emissions by 2050 (Bundesklimaschutzgesetz,^2^ Annex 2). Sectoral targets vary between 77% (energy) and 36% (agriculture) GHG emission reductions by 2030 (BMU 2016, Bundesklimaschutzgesetz). In addition to these emission reductions, Germany is targeting an increase in annual CO_2_ uptake from land use, land use change, and forestry (LULUCF) to 25 million tons CO_2_e by 2030 and 40 million tons in 2045 (Bundesklimaschutzgesetz). Even though the food and land use system in Germany is not forced to reduce emissions to the same extent as other sectors, doing so would alleviate pressure on these sectors and ensure an overall more rapid move towards achieving the goals set out in the Paris Agreement. In Germany, agriculture was directly responsible for 8.2% of all GHG emissions in 2020, not including overlap with other sectors such as energy requirements for fertilizer production. Around 50% of these emissions are methane emissions from enteric fermentation and manure management, 45.6% nitrous oxide emissions from fertilizer applications and manure management, and the rest are CO_2_ emissions from other management operations such as liming (Umweltbundesamt 2021).

If a nation’s entire food system with all relevant processes—growing, harvesting, processing, packaging, transportation, marketing, consumption, distribution, and disposal—is considered, emissions attributable to the food system can reach double the emissions from agriculture alone (Tubiello et al. 2021). Moreover, the food system is a major determinant of how agricultural area is managed in a country. For example, the amount of food lost post-harvest determines the surplus which has to be produced, and the consumption level of certain food groups determines the area dedicated to their production. As the production of meat-based proteins requires 30–40 times more area than the production of the same amount of cereal-based proteins (Poore and Nemecek 2018), the diet of a population is significantly influencing land use, not only domestically but also in countries meat is imported from. In food systems which evolve to require less agricultural area, it may be possible to change land use towards systems with higher carbon sequestration rates, which may reduce GHG emissions of up to a third of the amount stipulated in the Paris Agreement (Roe et al. 2019).

Future land use scenarios such as the one just described are important drivers of projections for socioeconomic and climate development (e.g. Riahi et al. 2016) and can also be used to study and assess various human–environment interactions beyond climate (e.g. Habel et al. 2019). Most scenarios are created by scientists and can be interpreted as possible futures relying on natural, technical, and economic concepts. However, the societal plausibility of the scenarios is usually not investigated: using the web of knowledge platform, we found more than 76 thousand studies on combined searches of the topics ‘land use’ and ‘pathways’. When we further restricted the search criterion to relate to the area of social sciences or the topic ‘plausibility’, the returned studies decreased by more than 99%. If, instead, the topic ‘potential’ was used as an additional search restriction, more than 20% of the originally found studies remained. This small experiment reveals an unfortunate separation between integrated scientific assessments and plausible political objectives. While integrated scientific assessments are generally more consistent, political objectives, on the other hand, are often compromises between different societal interest groups or between different regional entities. These societal compromises appear to be more plausible but may suffer from inconsistencies and inefficiencies. Inconsistencies may result from different institutions dealing with different policy objectives for shared resources. Inefficiencies can arise if theoretically promising pathways are societally contentious and compromises are needed to find a generally more acceptable but less efficient pathway. For example, many studies have shown meat consumption in general and beef specifically to be a key source for greenhouse gases from the food and land use sector. However, while a substantial reduction or outright ban of these products might be very efficient for climate protection, neither consumers nor farmers would support this. Even discussing policies far less stringent than an outright ban, such as a meatless ‘Veggie Day’, has fostered strong backlash in the population.

Deriving land use scenarios consistent with current or planned policies is usually straightforward. In Germany, planned and/or already implemented mitigation measures for agriculture include a 20% reduction of nitrogen fertilizer applications, an increase in the share of land farmed organically from currently 10 to 30% in 2030, the reduction of food waste by 25–50% and the promotion of more plant-based diets; other plans are more vague, such as the strengthening of the resilience of the food system (BMEL 2019; BMU 2020; Bundesregierung 2008). It is unclear, however, how close the suggested policies, by themselves and when bundled, will actually bring the food and land use system towards the targeted goals. It is also unclear how stakeholders view these plans and whether they consider them realistic or acceptable.

In this paper, we address these issues by first changing the angle and approaching the topic from a bottom-up instead of top-down perspective, and, second, by estimating the magnitude of change in the food and land use system under the suggested policies. For this, we survey different stakeholder groups on their opinion about realistic changes in the food and land use system in Germany up to 2050. Based on the answers, we develop four stakeholder pathways (a common stakeholder pathway, and three pathways representing stakeholder opinions from the academic, public and private sectors) and use an accounting tool to determine the effect of each stakeholder pathway on indicators such as land use, biodiversity conservation potential, food consumption, trade, and GHG emissions. Specifically, we examine if the changes stakeholders deem realistic for the German food and land use system by 2050 can (1) meet either of the various GHG reduction targets, (2) increase the area suitable for biodiversity conservation, and (3) increase the resilience of the food system. Furthermore, we compare the trajectories of these indicators under the stakeholder pathways to the trajectories under a pathway based on the trends we currently observe, and the trajectories under a pathway developed to portray a sustainable change in food and land use systems. By doing this, we can determine how far the stakeholder pathways take us away from the pathway of current trends and towards a pathway leading to a sustainable food and land use system, and how much more ambitious we may have to be in the future to meet our sustainability targets.

## Materials and methods

### Description of FABLE calculator

The FABLE calculator is developed and maintained by the FABLE consortium,^3^ a group consisting of a core-team and currently 20 country teams. It is an open-source Excel model that can be used to study the potential evolution of food and land use systems under different policies and socioeconomic development pathways. As an Excel-based tool, it is more accessible and easier to understand for non-modellers than other agroeconomic tools and allows for a deeper stakeholder engagement. Furthermore, every participating country develops their own bottom-up pathways, thus reflecting policies and discussions more realistically than what is possible in more generic agroeconomic models. The different pathways can be brought together in so-called ‘Scenathons’, where collective global impacts are calculated based on the different national calculators.

The FABLE calculator focuses on agriculture as the main driver of land use change and can be used as an accounting tool to depict changes in the level of agricultural activities, land use, food consumption, trade, and GHG emissions in each 5-year time step in the period 2000–2050. It includes 76 agricultural raw and processed products from the crop and livestock sectors and relies extensively on the FAOSTAT database^4^ for input data. Every country team has their own national FABLE calculator, as the input data on current diet, socioeconomic development status, population, agricultural and forest area share, etc. differ between countries.

To start a calculation, the user defines the pathway they want to analyse by selecting one of the available development scenarios for each of the 15 parameter items contained in FABLE, such as different population and GDP trajectories or shifts in food consumption (Table 1). At the core of these calculations are the scenario definitions, where shifters are calculated that implement the scenario target values over time by partially shifting the historic, usually 2010, values depending on the chosen implementation rate. The variety of available scenarios for each item ensures that the user can explore the potential impact of current policies or those that are not (yet) in place, and/or test for the consequences of a wide range of ‘if’ assumptions and their most important dependencies. As national targets may not be the same, the scenarios available for selection can differ between the different national FABLE calculators.

**Table 1:** Projections and scenarios the user may choose from before running a calculation with the German FABLE calculator. More scenarios can be added by the users

Item	Available scenarios	Description
GDP projections	SSP1, SSP2, SSP3	Speed of economic growth of advanced countries and speed of convergence for other countries
Population projections	SSP1, SSP2, SSP3, SSP4, SSP5, UN_medium, UN_high, UN_low, UN_constfertility, UN_instantreplacement, UN_momentum, UN_zeromigration, UN_constantmortality, UN_nochange	Fertility, mortality, migration, education, and urbanization rates
Alternative diets	SSP1, SSP2, SSP3, noChange, EATLancetAverage, 9050101p5, 8050202p0, FatDiet, NatHealthyDiet	Calorie demand per capita, share of plant-based calories, ratio of vegans and vegetarians, macronutrient distribution
Food waste	Current, Increased, Rapid50, Slow50	Share of food consumption which is wasted
Imports	I1, I2, I3	Share of consumption which is imported
Exports	E0, E1, E2, E3, Redux	Evolution of exports
Livestock productivity	NoGrowth, BAUGrowth, HighGrowth, LowGrowth	Productivity in 2050 in comparison to productivity 2000–2010
Crop productivity	NoGrowth, BAUGrowth, HighGrowth, LowGrowth	Productivity in 2050 in comparison to productivity 2000–2010
Agricultural expansion	NoExpansion, NoDefor2030, FreeExpansion	Agricultural area expansion
Afforestation	NoAffor, BonnChallenge	Afforestation
Activity of population	Low, Middle, High	Active or sedentary lifestyle of population
Climate change	NoChange and combinations of: RCPs: 2.6, 6.0 GCMs: hadgem2-es, gfdl-esm2m, ipsl-cm5a-lr, miroc-esm-chem, noresm1-m Crop models: GEPIC, LPJmL	Scenarios of climate change with or without crop productivity adjustment
Protected areas	NoChange, PAEExanpsion, PAAichi30, PAAichi50	Expansion of protected areas beyond current shares
Post-harvest losses	NoChange, Reduced	Share of supply lost during storage and transportation after 2010
Biofuel demand	NoChange, OECD_AGLINK, National	Biofuel demand until 2050

For each item, one scenario must be selected. The set of selected scenarios represents one pathway

After the selection of the scenarios, the calculator runs through a list of calculation steps (each comprising a large number of sub-calculations of which only the most central ones are introduced here) where all steps after the first are dependent on variables computed in the previous steps. In the first step, the targeted human consumption of different products is calculated based on historic data and scenario trajectories for population, diet, food waste, biofuel, and, for some diet scenarios, GDP, by first calculating demands for food, biofuels, and other non-food uses, then adding them up.

In steps two and three, these values form the base for the calculation of, first, the necessary livestock, and, taking crop requirements for livestock feed into account, crop production. Here, the scenarios on productivity growth, post-harvest loss, imports, and exports are taken into account. For livestock, herd sizes are calculated by adding exports to and subtracting imports from the domestic product demand, differentiating between different product sources (e.g. milk from dairy cattle, other cattle, or sheep and goats). Based on the herd sizes, historical feed requirements, and the productivity shifters, feed requirements per livestock and crop type are calculated. For livestock, an increasing productivity increases the required feed. From herd size and stocking rates, required pasture area is calculated. Similar to the livestock requirements, crop area is calculated based on the calculated demand from step one, the required feed, crop imports and exports, historic yield data, post-harvest loss, productivity growth, and the related scenarios.

In step four, the pasture and cropland area required for production as well as other land requirements, e.g. for planned afforestation, are calculated. This takes into account scenarios on allowed cropland expansion, protected area growth, and afforestation policies. In step five, it is checked if the required pasture and cropland area is higher than the maximum available area (i.e. total land minus urban, protected, and new forest area). If it is, the area (step six), livestock production (step seven), crop production (step eight), and human consumption (step nine) are reduced to feasible values based on the maximum available area. For example, if a slight cropland expansion would be required to fulfil all demands, but the expansion is not possible or allowed, the ratio between required and allowed cropland is calculated and used to downscale area and production for all crops equally, which further affects livestock numbers and final indicators. If crop and pasture area are below the available maximum, unused area is added to the other natural land category and the targeted values are accepted as the feasible values.

In the last step, indicators for food security, GHG emissions, biodiversity, freshwater use, self-sufficiency, and diversification are calculated based on the feasible consumption, production and land use values. The main indicators accounted for in the FABLE calculator consist of:

- Food (total kcal, g protein, g fat per capita per day)
- Biodiversity (protected areas per land cover type, area where natural processes predominate)
- Land (cropland area, pasture area, urban area, forest area, afforested area, other land area)
- GHG emissions (CO_2_, CH_4_, N_2_O emissions from crops, N_2_O, CH_4_ emissions from livestock, CO_2_ emissions from deforestation, CO_2_ emissions from other land conversion, CO_2_ sequestration from agricultural land abandonment, CO_2_ sequestration from active afforestation)
- Water (green, blue, grey water consumptive use for crops)
- Trade (import quantity, export quantity)
- Supply (production quantity, harvested area, planted area, crop yield, number of animals, production losses, biofuel production)
- Demand (feed consumption, food consumption, food waste, processing for biofuel, other processing, stock variation)

A detailed documentation of the FABLE calculator is provided in Mosnier et al. (2020).

### Description of stakeholder survey

We sent invitations to the survey to the main email addresses of the following 51 institutions: One to the German Federal Environmental Agency (Umweltbundesamt), one to the German Federal Office for Agriculture and Food (Bundesanstalt für Landwirtschaft und Ernährung), thirteen to state departments of agriculture (Landwirtschaftsministerien), seven to state chambers of agriculture (Landwirtschaftskammern), eleven to farmer’s associations (Bauernverbände), eleven to organizations of the private sector such as supermarket chains, lobby groups, and other associations, and seven to universities and research institutes. In each invitation, we asked that the email be forwarded to one or more appropriate respondents, who should answer on behalf of their organization. The respondents were advised to skip questions that they felt were outside their area of expertise. We received 25 responses. Twelve respondents stated that their organization was a scientific institution, seven said it was part of the public sector and six stated it was a private association. The respondents had a background in agriculture (16), climate (2), animal husbandry (2), environmental protection (2), soil science (1), communication (1), and nutrition and health (1).

To derive a stakeholder pathway for future food and land use systems, we asked the following questions:

1. By what percent should the consumption of animal fat, beef, cereals, chicken, eggs, fish, legumes, milk products, nuts, pork, potatoes, sugar, vegetable fat, and vegetables and fruits each change by 2050, respectively?
2. By what percent can we realistically decrease food waste by 2050?
3. Will the productivity of crop products (cereals, corn, legumes, potatoes, vegetables) strongly increase, slightly increase, stay constant, slightly decrease or strongly decrease by 2050, respectively?
4. Will the productivity of livestock products (beef, chicken, eggs, milk, pork) strongly increase, slightly increase, stay constant, slightly decrease or strongly decrease by 2050, respectively?
5. What percent of soy imports (for fodder) can be replaced with alternative protein sources by 2050?

### Scenario analysis with FABLE calculator

To properly assess the impacts of the stakeholder pathway on a future land use and food system in Germany, we compare it to two pathways that were already defined for a previous assessment (Steinhauser and Schneider 2020). The current trends pathway corresponds to a future based on current policy and historical trends (Table 2, ‘Current trends’). It is characterized by a small population decline (from 81.86 million inhabitants in 2020 to 78.91 million in 2050), significant constraints on agricultural expansion, no change in the extent of protected areas, medium productivity increases in the agricultural sector, and a 50% reduction of the share of food that is wasted by consumers over the period 2010–2050.^5^ Furthermore, we assume an evolution towards a slightly more flexitarian diet, expressed through a cultural shift towards 10% vegetarians and 1.5% vegans, as well as a reduction in average overall caloric intake by 10%.

**Table 2:** Scenario portfolios of the future food and land use pathways ‘current trends’, ‘sustainable’, and ‘stakeholder’

Scenario	Current trends	Sustainable	Stakeholder (4 pathways)
GDP projections	SSP2	SSP1	SSP2
Population projections	SSP2	SSP1	SSP2
Diets	90-50-10-1.5^a^	70-50-20-2.0^b^	StakeDiet (Table 3)
Food waste	Slow 50%^c^	Rapid 50%^c^	StakeLoss (20, 25, 20, 15%)
Import changes	I2 (stable)	I3^d^ (− 50%)	StakeImp (− 30, − 75, − 20, − 28%)
Export changes	E0 (stable)	Redux^e^ (− 25%)	E0 (stable)
Livestock productivity	BAUGrowth	HighGrowth	StakeGrowth (Fig. 1b)
Crop productivity	BAUGrowth	HighGrowth	StakeGrowth (Fig. 1a)
Climate change scenario^f^	RCP6p0	RCP2p6	RCP6p0
Post-harvest losses	No change	Reduced^g^	Reduced^g^
Protected areas	No change	Aichi50^h^	No change
Agricultural expansion	NoExpansion	NoExpansion	NoExpansion
Afforestation	NoAffor	NoAffor	NoAffor
Activity level of population^i^	Middle	Middle	Middle
Biofuel demand^j^	OECD_AGLINK	OECD_AGLINK	OECD_AGLINK

The listed scenarios were selected in the Scenario_definition Table of the FABLE calculator to run the calculation. In the stakeholder pathways, the scenarios ‘diet’, ‘food waste’, ‘import changes’ and ‘crop and livestock productivity’ were adapted to reflect the median responses, the academia responses, the public sector responses and the private sector responses. GDP, population and climate change projections followed the ‘current trends’ pathway

^a^90-50-10-1.5: By 2050, the diet of a German will be reduced to 90% of the total calories consumed in 2010 (FAOSTAT), sugar and fat consumption will be reduced by 50%, 10% of the population will be vegetarians, and 1.5% vegans

^b^70-50-20-2.0: By 2050, the diet of a German will be reduced to 70% of the total calories consumed in 2010 (FAOSTAT), sugar and fat consumption will be reduced by 50%, 20% of the population will be vegetarians, and 2% vegans

^c^Slow/Rapid 50%: Slow: 50% food waste share reached in 2050; rapid: 50% reached in 2030

^d^I3: imports of soy cake are reduced by 50% until 2050

^e^Redux: export of milk, pork and beef reduced by 25% until 2050

^f^Climate change scenario: GCM: hadgem2-es, crop model: GEPIC

^g^Reduced post-harvest losses: 50% reduction of crop and livestock products lost during storage and transportation compared to 2010

^h^Aichi50: expansion of protected areas by a minimum of 30% until 2030 and 50% until 2050

^i^Middle activity level: Moderately active lifestyle that includes physical activity equivalent to walking about 2.5–5 km per day at 5–6.5 km/h, in addition to the activities of independent living

^j^Biofuel demand based on OECD_AGLINK: projections of future biofuel demand until 2028 (OECD-FAO Agricultural Outlook), constant afterwards

The sustainable pathway represents a future in which significant efforts are made to adopt sustainable policies and practices (Table 2, ‘Sustainable’). In this future, the population grows slightly from 82.2 million in 2020 to 82.4 million in 2050 due to SSP1-associated improvements in overall human wellbeing and mortality. The extent of protected areas increases to 42% in 2050, there is a high productivity increase in the agricultural sector, the share of consumer food waste is reduced relatively quickly to 50% of the current share (the target value is reached by 2030 instead of 2050 and kept constant afterwards), and there is a stronger cultural shift towards a more flexitarian diet, with an increasing number of vegetarians (20%) and vegans (2%) by 2050, as well as a 30% reduction in overall average caloric intake. The sustainable pathway was developed by scientists of Universität Hamburg for the 2020 Report of the FABLE consortium (Steinhauser and Schneider 2020) in consultation with national stakeholders and experts.

Based on the survey and the already existing pathways, we defined four stakeholder pathways (Table 2). One pathway depicts a common vision amongst all stakeholders, one pathway is based on the responses of the scientists that answered the survey, one pathway on the responses of the public sector employees, and one pathway on the responses of members of private associations and organizations. Items of the pathways that were not covered by the survey, such as GDP, population, and climate change projections, were assumed to follow the same trends as in the 'current trends’ pathway.

## Results

In the following, we first describe the stakeholders’ responses to the online survey about realistic changes in the food and land use system in Germany by 2050 and how this translates into several stakeholder scenarios. Then, we examine if the stakeholder pathways can meet various GHG reduction targets (36%, 55%, or 65% by 2030), increase the area suitable for biodiversity conservation, and increase the resilience of the food system. For this, we assess the trajectory of different indicators in the stakeholder pathways, compare them to target values, and use a current trends and a sustainable pathway scenario to quantify the level of ambition inherent in the stakeholder pathways.

### Stakeholder opinions

The analysis of the answers to the stakeholder survey revealed that the different stakeholder groups from academia, the public and the private sector sometimes held strongly deviating opinions. Mostly members of academia advocate for zero consumption of animal products and believe that a higher reduction in food waste is realistic. Stakeholders from the private sector are more conservative in their estimations of food waste reduction and do not think that the consumption of animal products will decline by more than 30%. We thus decided to formulate four different stakeholder pathways: the first pathway is based on the median of all answers and represents a common stakeholder pathway, the other three pathways are based on the median responses of each stakeholder group and show the variety of opinions held in the German food and land use sector.

#### Q1—Changes in diet by 2050

We received 23 responses to the first question. Based on the whole dataset, the consumption of animal products should decrease by 2050, strongly for meat products (median − 50 to − 60%) and less strongly for fish, eggs, and milk products (median − 15 to − 30%), whereas the consumption of crop products should increase by at least 20% (Table 3, columns ‘%’). When looking at the different stakeholder groups, the envisioned changes in diet differ markedly, especially for animal products. People from academia envision a median reduction of 73% for beef and 65% for pork consumption (12 responses), whereas members of the private sector think a median reduction of 15% for beef and 30% for pork is more realistic (6 responses). Despite the different estimates for the future consumption of different food groups, the total calories consumed in each diet are in the same range of roughly 3000 kcal per person and day (Table 3, last row). To implement the stakeholder values as alternative diet scenarios in the FABLE calculator, we multiplied the calories consumed per product category in 2010 (FAOSTAT) with the percent change given by the stakeholders. The resulting values for kcal consumption per capita per day for each product group (Table 3, columns ‘kcal’) were entered into the FABLE calculators as the scenario ‘StakeDiet’.

**Table 3:** Summary of the diet change envisioned by the German stakeholders for the year 2050

	FAO2010	Common		Academia		Public sector		Private sector	
	kcal	kcal	%	kcal	%	kcal	%	kcal	%
Animal fat	258	155	− 40	71	− 73	181	− 30	239	− 8
Beef	47	24	− 50	13	− 73	24	− 50	40	− 15
Cereals	792	1029	+ 30	1089	+ 38	1069	+ 35	950	+ 20
Chicken	65	32	− 50	32	− 50	32	− 50	61	− 5
Eggs	47	37	− 20	26	− 45	37	− 20	47	0
Fish	37	31	− 15	22	− 50	31	− 15	37	0
Legumes	12	17	+ 45	18	+ 50	15	+ 30	14	+ 23
Milk products	296	207	− 30	163	− 45	222	− 25	267	− 10
Nuts	50	60	+ 20	60	+ 20	50	0	60	+ 20
Pork	259	104	− 60	91	− 65	104	− 60	181	− 30
Potatoes	104	125	+ 20	125	+ 20	125	+ 20	125	+ 20
Sugar (added)	394	256	− 35	197	− 50	275	− 30	266	− 33
Vegetable oils	420	504	+ 20	535	+ 28	462	+ 10	441	+ 5
Vegetables, Fruits	146	182	+ 25	208	+ 43	175	+ 20	179	+ 23
Alcohol	232	232	–	232	–	232		232	–
Beverages, Spices	32	32	–	32	–	32		32	–
Other	4	4	–	4	–	4		4	–
Sum	3194	3032		2917		3071		3175	

The column FAO2010 shows the average kcal consumption per capita and day in Germany in 2010 based on FAOSTAT. The next columns show for each stakeholder pathway the percentage change [%] given by the stakeholder groups in response to Q1: *By how many percent should the consumption of the listed products change by 2050*? and the resulting change in consumed kcal as used in the FABLE calculator [kcal]. The categories alcohol, beverages and spices and other were not surveyed

#### Q2—Realistic reduction of food waste until 2050

This question was answered by 23 respondents in total. The median of all responses was 20% (maximum 50%, minimum 5%). The median of the responses from people working in academia was 25% (11 responses), from the public sector 15% (7 responses) and from the private sector also 15% (5 responses). The values were added to the FABLE calculators as an option in the scenario selection for food loss share in 2050 (‘StakeLoss’). We assume that the reduction in food loss will commence linearly from 2010 to 2050, when the final value of each pathway is reached.

#### Q3—Changes in crop productivity by 2050

**Fig. 1:**
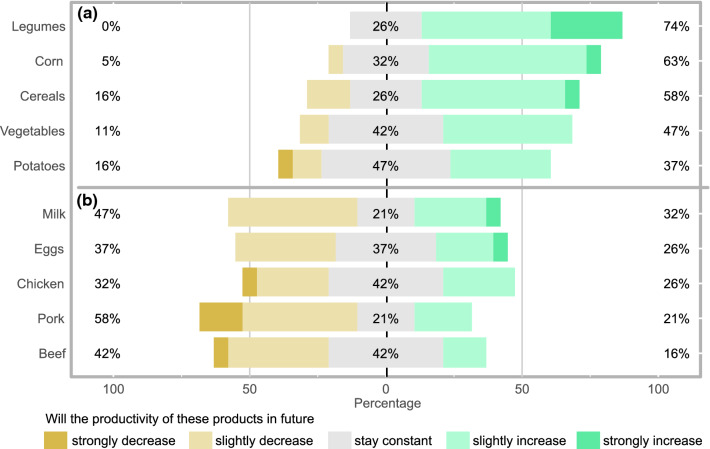
Stakeholder answers to the question if the productivity of **a** crop and **b** livestock products will decrease, increase or stay constant in 2050 in comparison to the productivity change observed in the historical time period 2000–2010. Percentages on the left show the share of responses assuming a slight or strong decrease in productivity growth, in the middle no change, and on the right a slight or strong increase

23 respondents answered this question. The majority of respondents agreed that the productivity growth of crop products will slightly increase in comparison to the productivity growth observed in the time period 2000–2010 (Fig. 1a). Only for potatoes does a majority think that the productivity growth will stay constant. Since there was a rather wide spread of opinions but no clear distinction between the different stakeholder groups, we decided to only consider the whole dataset and use the same scenario in each pathway. For the implementation into the FABLE calculators we had to assign values to the Likert items we offered as choices (strong increase, slight increase, constant, slight decrease, strong decrease). The crop productivity growth scenarios already implemented in FABLE consist of the scenarios NoGrowth, LowGrowth (lower growth rate than in the period 2000–2010), BAUGrowth (same growth rate as in the period 2000–2010) and HighGrowth (higher growth rate than in 2000–2010). We thus assigned the productivity multipliers as follows to the Likert items: strong increase = HighGrowth, slight increase = (HighGrowth + BAUGrowth)/2, constant = BAUGrowth, slight decrease = LowGrowth, strong decrease = (NoGrowth + LowGrowth)/2. The multipliers were then calculated as the weighted mean over all responses, with the weight being the ratio of respondents who selected a specific Likert item. The stakeholder multipliers for crop productivity changes fall between the BAUGrowth and HighGrowth multipliers. The adjusted growth scenario was added to the scenario selection as ‘StakeGrowth’.

#### Q4—Changes in livestock productivity by 2050

19 respondents answered this question. Depending on the type of livestock, 32–58% of respondents agreed that livestock productivity will decrease in future, yet a considerable number of respondents also think that it will stay constant (21–42%) or even increase (16–32%) (Fig. 1b). The implementation into FABLE followed the same steps as described in Q3 above. The calculated stakeholder multipliers in the ‘StakeGrowth’ scenario for livestock productivity fall between the LowGrowth and BAUGrowth multipliers.

#### Q5—Reduction of soy imports by 2050

17 respondents answered this question. The median response was that 30% of soy imports may be substituted with other protein sources by 2050 (maximum 100%, minimum 2%). The median of the responses from people working in academia was 75% (8 responses), from the public sector 28% (6 responses) and from the private sector 20% (3 responses). We implemented these values into the FABLE calculators by specifying a linear reduction in soy cake import rates until the total reduction specific to each pathway is reached in 2050 (‘StakeImp’).

### Pathway analysis: land and biodiversity

#### Status quo

In 2010, Germany was covered by 36% cropland, 14% grassland, 32% forest, 7% urban, and 11% other natural land. The ‘other land’ category covers the remaining terrestrial area not covered by the categories above and can be very heterogeneous, including degrees of wilderness. In 2020, land where natural processes predominate accounted for 19% of Germany’s terrestrial land area. The term covers ‘areas where natural processes predominate, but are not necessarily places with intact natural vegetation, ecosystem processes or faunal assemblages’ (Jacobson et al. 2019), and is an indicator related to biodiversity conservation.

#### Projections

**Fig. 2:**
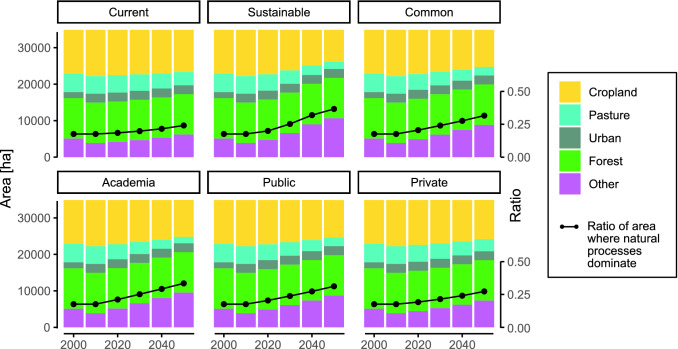
Evolution of area by land cover type in the current trends, sustainable and stakeholder pathways. The black lines in the plots denote the ratio of area where natural processes predominate

Land cover changes from 2030 to 2050 will be dominated by a decrease in pasture and an increase in other land area in the current trends pathway (Fig. 2). The reduction in pasture is a consequence of the increase in livestock productivity per head, the stable livestock density, and the decrease in red meat consumption. Even though milk consumption increases over time slightly in the current trends pathway, this increase is compensated by the other dynamics. The ratio of areas where natural processes predominate rises from 19% in 2020 to 24% in 2050, denoting only a small benefit for biodiversity conservation and protection. In the sustainable pathway, the expansion of protected areas by a minimum of 30% until 2030 and 50% until 2050 is mandated, and dietary shifts towards a healthier diet with lower calories, and a lower consumption of pasture-intensive animal products is assumed, which explains the more pronounced decrease in pasture area and the decrease in cropland area in comparison to the current trends pathway. The share of land where natural processes predominate rises to almost 37%. The dynamics in the common stakeholder pathway fall in the middle between the current trends and the sustainable pathway: the reduction of pasture and cropland area and increase in other land area are not as pronounced as in the sustainable pathway, yet higher than in the current trends pathway. The share of land where natural processes predominate rises to 32%. The pathway envisioned by only the members of academia would lead to a land use change more aligned with the sustainable pathway, with a reduction of cropland share from 36% in 2010 to 29% in 2050, and a reduction of pasture share from 14 to 5%. The share of land where natural processes predominate rises to 33%. The land use changes observed for the pathways developed from the expert opinions of stakeholders in the public and private sectors are similar in terms of cropland expansion—the shares in 2050 are 29% and 31% in the public and private pathways, respectively—but deviate in terms of pasture area, which only declines to 7% in the public and 9% in the private sector pathway. The share of land where natural processes predominate rises to 31% (public sector pathway) and 27% (private sector pathway).

### Pathway analysis: GHG emissions from AFOLU

#### Status quo

Direct GHG emissions from Agriculture, Forestry, and Other Land Use (AFOLU) accounted for 54.8 TgCO_2_e in Germany in 2021 (preliminary estimation), which is 7% of total emissions (Umweltbundesamt 2021). Enteric fermentation is the primary source of AFOLU emissions (56.4%), followed by fertilizer applications and manure management (38.8%), as Germany has a large number of dairy cattle, manure storage units, and practice-intensive application of manure and other fertilizers. Furthermore, Germany’s agricultural land has a considerable share of drained and degraded peatlands, which are often not only very productive, but also sources of a significant amount of GHG emissions (Tubiello et al. 2016).

#### Projections

**Fig. 3:**
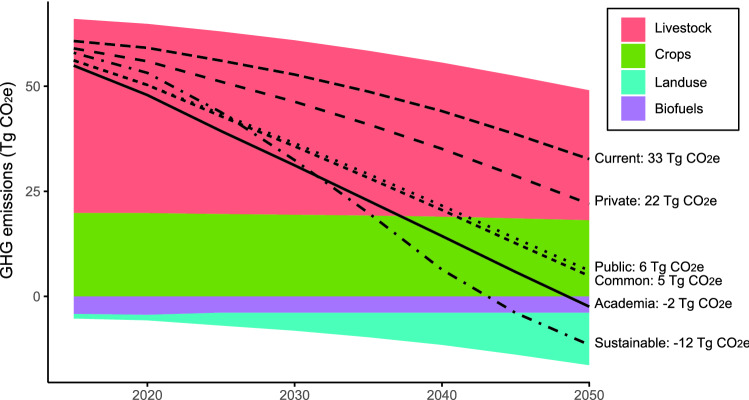
Potential AFOLU emission trends until 2050 by land use category in the current trends pathway. The lines show estimated total emission trends in the different pathways

Under the current trends pathway, total agricultural GHG emissions decline from 2010 to 2050 by 56% to 33 TgCO_2_e (Fig. 3). Emissions from cropland stay nearly constant over that time, but emissions from livestock go down by 33% to 31 TgCO_2_e in 2050, and there are negative emissions from changes in land use of − 13 TgCO_2_e (Table 4). The decline in emissions from livestock is explained by the assumed increase in livestock productivity per head, the stable livestock density, and the decrease in red meat consumption in the current trends pathway. The negative emissions stem from the transformation of managed land (cropland, pasture) to ‘other land’, where sequestration of carbon is higher. In the sustainable pathway, total emissions from crops, livestock, biofuels and land use drop to − 12 TgCO_2_e. In the four stakeholder pathways, the decrease of total emissions is variable. While the changes in the food and land use system in the common and the public sector stakeholder pathways cause a potential decline of emissions to 5 and 6 TgCO_2_e, respectively, the private sector stakeholder pathway may lead to total emissions in the amount of 22 TgCO_2_e, and the academia stakeholder pathway to negative emissions of − 2 TgCO_2_e. With an emission reduction goal for the German agricultural sector of 36% in 2030 compared to emissions in 1990 (76.5 Tg CO_2_e), the current pathway will not quite reach this goal with a reduction of only 31%. The sustainable and all four stakeholder pathways, however, will surpass this goal and even reach or almost reach the EU’s emission goal of 55% (Table 4, second to last column). None of the pathways will achieve Germany’s general emission reduction goal of 65% in 2030. Going further in time, the sustainable and the academia stakeholder pathways may achieve negative emissions between 2040 and 2050, while the common and public sector stakeholder pathways almost but not quite reach net-zero emissions by 2050.

**Table 4:** Total CO_2_ emissions (in Tg CO_2_e) by source in the reference year 1990 (Rösemann et al. 2021) and estimated for 2030 and 2050 under the different pathways

	Livestock		Crops		LUC		Biofuels		Total	
Tg CO_2_e 1990:	52.3		24.2		–		− 0.9 (2000)		76.5	
Tg CO_2_e in:	2030	2050	2030	2050	2030	2050	2030	2050	2030	2050
Current trends	41.5	30.9	19.4	18.1	− 4.3	− 12.5	− 3.9	− 3.9	52.8 (− 31%)	32.7 (− 57%)
Sustainable	33.9	16.5	17.1	12.3	− 14.7	− 36.4	− 3.9	− 3.9	32.4 (− 58%)	− 11.5 (− 115%)
Common	34.1	20.5	17.8	14.9	− 12.4	− 26.7	− 3.9	− 3.9	35.7 (− 53%)	4.9 (− 94%)
Academia	31.9	17.0	17.7	14.8	− 14.6	− 30.3	− 3.9	− 3.9	31.2 (− 59%)	− 2.4 (− 103%)
Public	34.4	21.0	17.9	15.1	− 12.0	− 26.1	− 3.9	− 3.9	36.4 (− 52%)	6.1 (− 92%)
Private	39.1	28.1	18.6	16.4	− 7.5	− 18.6	− 3.9	− 3.9	46.3 (− 39%)	22.0 (− 71%)

The percentage change values of total CO_2_ emissions in the last two columns refer to the changes between 1990 and 2030/2050

The difference between the total emission reductions calculated for the different pathways can be mainly attributed to a higher sequestration due to land use change, followed by a reduction in the livestock sector and only marginally to a decrease in the emissions of crop production (Table 4). There is a considerable difference in AFOLU GHG emission estimations between the stakeholder pathways, which is most likely caused by the different changes in diet with a much higher reduction of animal product consumption in the academia and public sector pathways than in the private sector pathway. The projected crop and livestock productivities are the same in all stakeholder pathways and therefore cannot contribute to the observed difference in emissions. Lastly, the amount of food waste reduction also does not seem to contribute noticeably to the amount of GHG emissions. The stakeholders deemed a food waste reduction of 15–25% until 2050 as realistic, whereas in the current and sustainable pathways, it is assumed that food waste will be reduced by 50% in the same time horizon. Considering that food waste contributes significantly to GHG emissions (Adelodun and Choi 2020; MacRae et al. 2013), it is unexpected to see that our results are not as sensitive to the level of reduction as may have been assumed.

### Pathway analysis: diet and food security

#### Status quo

In Germany, undernutrition is negligible (FAO et al. 2020), but 54% of adults were overweight and 18% obese in 2014 (Robert Koch Institut 2018; Schienkiewitz et al. 2017). Based on the reference system of the International Obesity Task Force, 19.3% of children between age 3 and 17 were overweight between 2014 and 2017. This is a minute decline since the 2010 baseline study (Schienkiewitz et al. 2018). In 2017, approximately 7–10% of the population suffered from diabetes type 2, numbers which are projected to increase even further in the coming decades (Tönnies et al. 2019). In 2014/15, about 6% of all German adults suffered from coronary heart disease (Busch and Kuhnert 2017), and cardiovascular diseases are the primary cause of death with about 40% (Dornquast et al. 2016), which can be attributed in part to dietary risks (Mozaffarian 2016). The main source of daily calories comes from crop products (ca 1500 kcal/person), followed by animal products (ca 1000 kcal/person) and other products such as sugar, alcoholic and non-alcoholic beverages (ca 630 kcal/person). Concerning the provision of food, the agricultural sector of Germany is reliant on imports for a variety of products, especially feed for livestock. Much is imported from European neighbours, which makes Germany not strictly self-sufficient, but still resilient in terms of food security. In 2010, self-sufficiency was only achieved for the product groups cereals, milk and dairy, oilseeds and vegetable oils, pork, and roots and tubers.

#### Projections

**Fig. 4:**
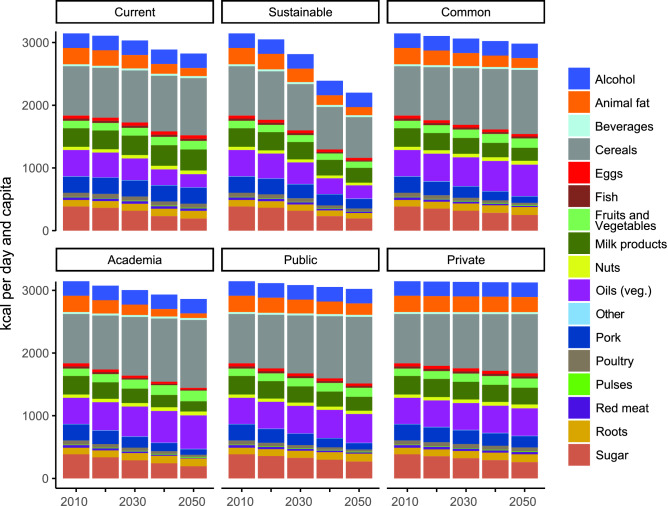
Projections of the average daily kilocalorie intake of different food groups under the pathways current trends, sustainable and stakeholder (common, academia, public and private sector)

In the current trends diet, the consumption of total calories is reduced by about 290 kcal per person per day by 2050. This reduction is mainly caused by a decrease in consumption of sugar, animal fat and vegetable oils (Fig. 4). In the sustainable diet, there is a decrease in oil and sugar consumption and in the consumption of all animal-based food groups, whereas the consumption of the other food products stays relatively constant. By 2050, this leads to an overall reduction in daily calorie intake of about 850 kcal per person. In the common stakeholder diet, the consumption of animal products is reduced by 38%, but the consumption of crop products is increased by 30% by 2050, which means that the total calories consumed per person per day stays relatively constant over time and there is only a slight decrease of 162 kcal per person per day from 2010 to 2050. When broken down to the different stakeholder groups, the diet shifts differ in the severity, but not the direction of change. In all pathways, there is a shift from animal-based to plant-based calories, but stakeholders in the academic sector envision a more radical change in comparison to the stakeholders of the private sector, with the public sector stakeholders in between. The total calories consumed per person per day decrease by 277, 123 and 19 kcal per person per day in the academia, public, and private diets, respectively, and thus stay below the reductions reached in the sustainable and even the current trends diet.

**Fig. 5:**
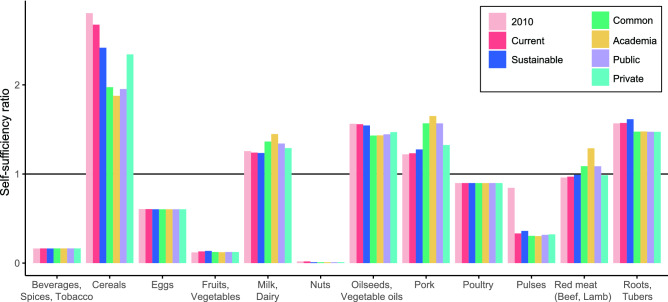
Self-sufficiency per product group in 2010 and 2050. Self-sufficiency is defined as the ratio of total internal production over total internal demand. A country is self-sufficient for a product if the self-sufficiency ratio of the product is equal to one

Concerning food provision, in all pathways, the dependency on imports for nuts, fruits and vegetables, beverages, spices and tobacco, pulses, eggs and poultry to satisfy demand remains unchanged (Fig. 5). If Germany was self-sufficient in 2010, as for cereals, the self-sufficiency ratio may decline in future, but not below the self-sufficiency line. These projections show that despite changes in internal demand due to changes in diet, changes in crop and livestock productivity, and changes in food losses, the overall self-sufficiency status of Germany in regards to different product groups does not change.

## Discussion

In this study, we conducted a bottom-up analysis of a future food and land use system pathway for Germany. For this, we surveyed stakeholders in the food and land use system, evaluated their assessments, and created a pathway that we could analyse with the FABLE calculator. We analysed whether the stakeholder pathways could meet various GHG emission reduction targets, increase the area suitable for biodiversity conservation, and increase the resilience of the food system. We also compared it to a pathway following current trends and a pathway towards a sustainable food and land use system.

The results indicate that it will be possible to reach the German emission reduction goal for the agricultural sector of 36% by 2030 and come close to the general EU emission reduction goal of 55% under all stakeholder pathways. It is also possible to nearly reach carbon neutrality by 2050 in three of the four stakeholder pathways, with the private sector pathway being the exception. The two main factors for these AFOLU emission reductions are a diet-change-induced decrease in livestock production and, as a direct result of this, a decrease in agricultural land area for grazing and crops, which makes room for other land types with higher carbon sequestration rates. This finding is in accordance with other studies that have identified land use/land use change and dietary choices and consumption patterns as large contributors to reductions in GHG emission (Crippa et al. 2021; Dalin and Outhwaite 2019; Hayek et al. 2021; Willett et al. 2019). It should be mentioned that due to current German policies, no afforestation is allowed in the FABLE calculator when land use change occurs. All freed up agricultural area is instead shifted to ‘other natural land’. If an afforestation policy was implemented, parts or all of this area could be turned into forest, leading to even higher carbon sequestration.

The resilience of the food system in Germany is neither affected negatively nor positively in the stakeholder pathways, despite changes in food demand, changes in crop and livestock productivity, and changes in food losses. Concerning land suitable for biodiversity protection, we estimate that the area where natural processes predominate will rise from 19 to 27–33% under the stakeholder pathways, which is again mostly due to a change in diet and a reduction of livestock production. Crenna et al. (2019) also estimate that, in the EU, it is the meat products that have the most profound negative impacts on biodiversity. It remains unclear how valuable this area will be for biodiversity protection, as land only recently converted from intensive or extensive use to areas of low human impact does not have the same diversity and habitat quality as undisturbed areas with natural intact vegetation. These areas are usually the ones that host the highest amount of biodiversity (Green et al. 2005). However, even though areas with low human impact are not wilderness, they can help to slow down the loss of diversity and provide valuable ecosystem services (Jacobson et al. 2019).

Overall, our analysis of the different stakeholder pathways shows that all of them are leading towards a more sustainable food and land use system in Germany by 2050. It should be kept in mind that we used the ‘middle of the road’ (SSP2) scenario for GDP and population projections and the more pessimistic projections for climate change impacts on yield as contained in the current trends pathway. Had we used the SSP1 projections and the more optimistic climate change impact projections as in the sustainable pathway, the changes would have been even more pronounced. Many of the positive changes in the indicators can be attributed to a decreased production of animal products, mostly triggered by a change in diet. Yet, how realistic is it for the German population to change their diet as drastically as it was deemed realistic by many of the stakeholders, especially in academia and the public sector, with decreases in sugar and livestock product consumption of up to 70%? Globally, trends lead into the opposite direction, towards diets high in energy and low in micronutrient content, with increasing shares of meat, sugar, and processed foods (Caballero and Popkin 2002; Monteiro et al. 2010; Popkin and Nielsen 2003). In Germany, animal products contribute on average 35% to the daily calorie intake, a value which has only decreased slightly in the last years (Bundesinformationszentrum Landwirtschaft 2021). Even in our small sample of stakeholders, there was a very wide range of individual opinions, with some thinking that overall animal protein consumption may further increase in future, whereas others saw a steep rise in vegetarians and vegans. Based on these facts, it seems unrealistic that the stakeholders' envisioned drastic changes in diet may be realized, especially those by the academic and public sector stakeholders. However, other stakeholder groups have indicated a high amount of faith in the power of education (Garcia-Gonzalez and Eakin 2019) and health-promoting policies (Muller et al. 2009) to realize a change in consumption patterns; and believe that projects like community gardening and urban agriculture could promote a change in diet (Campbell 2004). Based on these assessments, the change in diet in the different stakeholder pathways may still be considered feasible, more so because they are not accompanied by a reduction in total calorie consumption, only a substitution of calorie sources.

Many previous integrated assessments of land use and food system developments have focused on natural, technical, and economic capacities. These assessments ignore social plausibility and acceptance. Conversely, political targets synthesize many societal dialogues between different interest groups, often at the expense of feasibility and consistency. This study tries to reconcile the strengths and weaknesses of scientific assessment and societal/political discourse to derive plausible futures of land use and food systems, which are acceptable, consistent, and feasible. One disadvantage of the online survey approach we used (due to COVID-19 restrictions) is that the answers could only be collected individually and not discussed in group settings to form a consensus. For this, a further Delphi survey (cf. Rayens and Hahn 2000) or a comparable approach would have been needed. Fortunately, other organizations in Germany such as the DAFA (German alliance for agricultural research) are in the process of developing goals for the agricultural sector using stakeholder working groups and meetings,^6^ from which further (consensus) scenarios for stakeholder analyses may be derived in future.

## Footnotes

1 https://ec.europa.eu/info/strategy/priorities-2019-2024/european-green-deal/delivering-european-green-deal_en.

2 https://www.gesetze-im-internet.de/ksg/.

3 http://www.foodandlandusecoalition.org/fable.

4 https://www.fao.org/faostat/en/#data.

5 Example: In 2010, approximately 12% of fruits and vegetables were discarded by consumers in Germany. The target is that by 2050, only 6% of fruits and vegetables should be thrown away.

6 https://www.dafa.de/landwirtschaft-2049/.

## References

[CR1] Adelodun B, Choi KS (2020). Impact of food wastage on water resources and GHG emissions in Korea: a trend-based prediction modeling study. J Clean Prod.

[CR2] BMEL (2019) Ackerbaustrategie 2035 - Perspektiven für einen produktiven und vielfältigen Pflanzenbau. Berlin, p 68

[CR3] BMU (2016) Klimaschutzplan 2050 - Klimaschutzpolitische Grundsätze und Ziele der Bundesregierung. Berlin, p 92

[CR4] BMU (2020) Wir schafft Wunder - Fortschritt sozial und ökologisch gestalten. Berlin, p 48

[CR5] Bundesinformationszentrum Landwirtschaft (2021) Bericht Markt- und Versorgungslage Fleisch. In: Bundesanstalt für Landwirtschaft und Ernährung. https://www.ble.de/DE/BZL/Daten-Berichte/Fleisch/fleisch_node.html. Accessed 14 Sept 2021

[CR6] Bundesregierung (2008) Deutsche Anpassungsstrategie an den Klimawandel (DAS). Berlin, p 78

[CR7] Busch MA, Kuhnert R (2017). 12-Monats-Prävalenz einer koronaren Herzkrankheit in Deutschland. J Health Monit.

[CR8] Caballero B, Popkin BM (2002). The nutrition transition: diet and disease in the developing world.

[CR9] Campbell MC (2004). Building a common table—the role for planning in community food systems. J Plan Educ Res.

[CR10] Crenna E, Sinkko T, Sala S (2019). Biodiversity impacts due to food consumption in Europe. J Clean Prod.

[CR11] Crippa M, Solazzo E, Guizzardi D, Monforti-Ferrario F, Tubiello FN, Leip A (2021). Food systems are responsible for a third of global anthropogenic GHG emissions. Nat Food.

[CR12] Dalin C, Outhwaite CL (2019). Impacts of global food systems on biodiversity and water: the vision of two reports and future aims. One Earth.

[CR13] Dornquast C, Kroll LE, Neuhauser HK, Willich SN, Reinhold T, Busch MA (2016). Regional differences in the prevalence of cardiovascular disease. Dtsch Arztebl Int.

[CR14] European Comission (2019) The European Green Deal. Brussels, p 24

[CR15] FAO, IFAD, UNICEF, WPF, WHO (2020) The State of Food Security and Nutrition in the World 2020. Transforming food systems for affordable healthy diets. FAO, Rome, p 320

[CR16] Garcia-Gonzalez J, Eakin H (2019). What can be: stakeholder perspectives for a sustainable food system. J Agric Food Syst Community Dev.

[CR17] Green RE, Cornell SJ, Scharlemann JPW, Balmford A (2005). Farming and the fate of wild nature. Science.

[CR18] Habel JC, Rasche L, Schneider UA (2019). Final countdown for biodiversity hotspots. Conserv Lett.

[CR19] Hayek MN, Harwatt H, Ripple WJ, Mueller ND (2021). The carbon opportunity cost of animal-sourced food production on land. Nat Sustain.

[CR20] Jacobson AP, Riggio J, Tait AM, Baillie JEM (2019). Global areas of low human impact (‘Low Impact Areas’) and fragmentation of the natural world. Sci Rep.

[CR21] MacRae R, Cuddeford V, Young SB, Matsubuchi-Shaw M (2013). The food system and climate change: an exploration of emerging strategies to reduce GHG emissions in Canada. Agroecol Sust Food.

[CR22] Monteiro CA, Levy RB, Claro RM, de Castro IRR, Cannon G (2010). Increasing consumption of ultra-processed foods and likely impact on human health: evidence from Brazil. Public Health Nutr.

[CR23] Mosnier A, Penescu L, Perez-Guzman K (2020). Documentation FABLE calculator 2020 update.

[CR24] Mozaffarian D (2016). Dietary and policy priorities for cardiovascular disease, diabetes, and obesity a comprehensive review. Circulation.

[CR25] Muller M, Tagtow A, Roberts SL, Macdougall E (2009). Aligning food systems policies to advance public health. J Hunger Environ Nutr.

[CR26] Poore J, Nemecek T (2018). Reducing food’s environmental impacts through producers and consumers. Science.

[CR27] Popkin BM, Nielsen SJ (2003). The sweetening of the world’s diet. Obes Res.

[CR28] Rayens MK, Hahn EJ (2000). Building consensus using the policy Delphi method. Policy Polit Nurs Pract.

[CR29] Riahi K, Van Vuuren DP, Kriegler E (2016). The shared socioeconomic pathways and their energy, land use, and greenhouse gas emissions implications: an overview. Glob Environ Change.

[CR30] Robert Koch Institut (2018). Kindliche Adipositas: Einflussfaktoren im Blick.

[CR31] Roe S, Streck C, Obersteiner M (2019). Contribution of the land sector to a 1.5 C world. Nat Clim Change.

[CR32] Rösemann C, Haenel H-D, Vos C (2021). Calculations of gaseous and particulate emissions from German agriculture 1990–2019: report on methods and data (RMD).

[CR33] Schienkiewitz A, Mensink G, Kuhnert R, Lange C (2017). Übergewicht und Adipositas bei Erwachsenen in Deutschland. J Health Monit.

[CR34] Schienkiewitz A, Damerow S, Rosario AS (2018). Prevalence of underweight, overweight and obesity among children and adolescents in Germany. KiGGS Wave 2 results according to international reference systems. J Health Monit.

[CR35] Steinhauser J, Schneider U, Poncet J (2020). Pathways to sustainable land-use and food systems in Germany by 2050. Pathways to sustainable land-use and food systems.

[CR36] Tönnies T, Röckl S, Hoyer A (2019). Projected number of people with diagnosed type 2 diabetes in Germany in 2040. Diabet Med.

[CR37] Tubiello FN, Biancalani R, Salvatore M, Rossi S, Conchedda G (2016). A worldwide assessment of greenhouse gas emissions from drained organic soils. Sustain Basel.

[CR38] Tubiello FN, Rosenzweig C, Conchedda G (2021). Greenhouse gas emissions from food systems: building the evidence base. Environ Res Lett.

[CR39] Umweltbundesamt (2021) Beitrag der Landwirtschaft zu den Treibhausgas-Emissionen. In: Umweltbundesamt. https://www.umweltbundesamt.de/daten/land-forstwirtschaft/beitrag-der-landwirtschaft-zu-den-treibhausgas#treibhausgas-emissionen-aus-der-landwirtschaft. Accessed 22 Dec 2021

[CR40] Willett W, Rockström J, Loken B (2019). Food in the Anthropocene: the EAT–Lancet Commission on healthy diets from sustainable food systems. Lancet.

